# Population subgroup differences in the use of a COVID-19 chatbot

**DOI:** 10.1038/s41746-021-00405-8

**Published:** 2021-02-19

**Authors:** Laura C. Schubel, Deliya B. Wesley, Ethan Booker, John Lock, Raj M. Ratwani

**Affiliations:** 1 MedStar Health National Center for Human Factors in Healthcare, Washington, DC USA; 2 MedStar Health Research Institute, Hyattsville, MD USA; 3 MedStar Telehealth Innovation Center, Washington, DC USA; 4 MedStar Health, Columbia, MD USA

**Keywords:** Health services, Public health

## Abstract

COVID-19 chatbots are widely used to screen for symptoms and disseminate information about the virus, yet little is known about the population subgroups that interact with this technology and the specific features that are used. An analysis of 1,000,740 patients invited to use a COVID-19 chatbot, 69,451 (6.94%) of which agreed to participate, shows differences in chatbot feature use by gender, race, and age. These results can inform future public health COVID-19 symptom screening and information dissemination strategies.

Chatbots, or conversational agents, are software programs that communicate with people through text or voice, and have emerged as a method to screen for, and disseminate heath information about, COVID-19 (refs. ^1,2^). Chatbots are being used by federal organizations like the Centers for Disease Control and Prevention, as well as healthcare facilities and other stakeholders, given the low cost of use, the ability to scale to reach tens of thousands of people, and the ability to reach people through commonly used modalities such as computers, mobile phones, and other devices^3^. Chatbot use is not limited to the general public, healthcare facilities are using chatbots as a way to rapidly screen healthcare workers^4^, assist patients with self-managing lifestyle choices and chronic conditions^5–7^, and reduce stigma during diagnosis and treatment^8^.

Engagement with health information through different technologies, like chatbots, can vary by patient demographics^9^. For example, in a controlled simulation study in which participants viewed patient COVID-19 screening with a chatbot vs. human, certain population subgroups viewed the chatbot as having greater abilities and benevolence than other population subgroups^10^. Despite the widespread use of COVID-19 chatbots, little is known about end-user interaction with chatbots and questions have been raised about clinical, legal, ethical aspects of this technology^11^.

We analyzed demographic and interaction data from over one million people who were sent a text message or email invitation to use a COVID-19 focused chatbot that provided a symptom screener and learning module. Our analysis aimed to understand interaction rates and features accessed by different population subgroups. Knowing this information could better inform public health information dissemination strategies for COVID-19 and could inform management of other health conditions.

Across all invited patients, the overall interaction rate was 6.94% (69,451 of 1,000,740 invited patients) and the mean and median patient age for those that engaged were 49.74 and 51 years, respectively (std 16.40). Of the patients that interacted with the chatbot, there was no difference in symptom screener use (*n* = 20,553, 29.59% of 69,451) compared to learning module use (*n* = 20,532, 29.56% of 69,451).

There were differences in chatbot interaction and module use by subgroup (Table 1). A higher proportion of female patients (7.68%, 95% CI 7.61, 7.75) interacted with the chatbot than males (5.91%, 95% CI 5.84, 5.98) (*p* < 0.0001), with greater use of the learning module (30.73%, 95% CI 30.30, 31.15) than males (27.45, 95% CI 26.89, 28.01) (*p* < 0.0001). A higher proportion of African American patients (7.58%, 95% CI 7.49, 7.67) interacted with the chatbot than white (6.54%, 95% CI 6.46, 6.61) (*p* < 0.0001), Hispanic or Latino patients (6.07%, 95% CI 5.85, 6.30) (*p* < 0.0001), or Asian American patients (6.64%, 95% CI 6.28, 7.00) (*p* < 0.0001), with greater use of the learning module (33.67%, 95% CI 33.09, 34.25) than white (27.64%, 95% CI 27.12, 28.16) (*p* < 0.0001), Hispanic or Latino (23.77%, 95% CI 22.16, 25.37) (*p* < 0.0001), and Asian American patients (24.49, 95% CI 22.09, 26.90) (*p* < 0.0001), and greater use of the symptom screener (35.42%, 95% CI 34.83, 36.01) than white (25.66%, 95% CI 25.15, 26.16) (*p* < 0.0001), Hispanic or Latino (23.95%, 95% CI 22.34, 25.56) (*p* < 0.0001), and Asian American (24.57, 95% CI 22.17, 26.98) (*p* < 0.0001) patients. Lastly, a higher proportion of patients aged 51–90 (8.86%, 95% CI 8.77, 8.95) interacted with the chatbot than patients aged 18–50 (5.67%, 95% CI 5.61, 5.73) (*p* < 0.0001) with greater symptom screener use by 18–50 year-olds (31.22%, 95% CI 30.72, 31.71) compared to 51–90 year-olds (28.03% 95% CI 27.56, 28.50) (*p* < 0.0001) and greater learning module use by 51–90 year-olds (34.21%, 95% CI 33.72, 34.71) compared to 18–50 year-olds (24.74%, 95% CI 24.29, 25.20) (*p* < 0.0001).

**Table 1:** Demographics of patients that were invited and used the chatbot with separation of module use.

	Invited (% of total invited)	Engaged (% of subgroup)	Symptom screener use (% of subgroup)	Learning module use (% of subgroup)
Sex				
Female	583,235 (58.28%)	44,784 (7.68%)	13,243 (29.57%)	13,761 (30.73%)
Male	416,905 (41.66%)	24,632 (5.91%)	7301 (29.64%)	6762 (27.45%)
Unknown	600 (0.06%)	35 (5.83%)	9 (25.71%)	9 (25.71%)
Race				
White	440,551 (44.02%)	28,792 (6.54%)	7387 (25.66%)	7958 (27.64%)
African American	335,687 (33.54%)	25,436 (7.58%)	9009 (35.42%)	8565 (33.67%)
Hispanic or Latino	44,676 (4.46%)	2714 (6.07%)	650 (23.95%)	645 (23.77%)
Asian American	18,512 (1.85%)	1229 (6.64%)	302 (24.57%)	301 (24.49%)
Other	161,314 (16.12%)	11,280 (6.99%)	3205 (28.41%)	3063 (27.15%)
Age				
18–50	601,685 (60.12%)	34,101 (5.67%)	10,645 (31.22%)	8438 (24.74%)
51–90	399,055 (39.88%)	35,350 (8.86%)	9908 (28.03%)	12,094 (34.21%)

Analysis of COVID-19 chatbot data reveals non-obvious differences in engagement and use of specific modules by population subgroup. In our patient population, a higher proportion of women, African Americans, and those aged 51–90 interacted with the chatbot, and used the learning module more than the symptom screener compared to their respective subgroup comparator. Interestingly, the rate of COVID-19-positive cases by race and gender during this time period mirrored the differences in chatbot interaction: 38.3% of positive cases were African American compared to 31.9% white and 53.8% were female compared to 46.2% male. These differences may partially explain the chatbot use results. Reliable age data for COVID-19-positive patients were not available.

The differences in chatbot interaction and use of the learning module and symptom screener, combined with research showing that patients are more likely to disclose health information when engaging with chatbots compared to humans, can inform COVID-19 screening and information dissemination strategies^12^. Patient education and patient reporting of information for COVID-19 screening can be maximized by using chatbots with those subgroups that are more likely to engage and using different methods for patients with lower engagement rates. Additional research is required to understand why these subgroup differences exist.

One limitation of this study is that a small percent of the total number of patients invited to use the chatbot elected to participate. This may partially be due to the healthcare system having inaccurate patient contact information for some patients resulting in unsuccessful invitations to use the chatbot. The chatbot was only available in English which may account for less engagement from Hispanic populations or other populations that do not speak English. Other limitations include not knowing if patients who initiated use of the modules completed them, and the potential for inaccurate patient demographic information in the electronic health record (EHR). These results may not be generalizable since they are based on a patient population that is predominantly black and white from a single healthcare system with most patients speaking English.

## Methods

### Chatbot data

Data were analyzed from a large healthcare system on the East coast of the United States with patient demographics that were:

- Gender: 55.6% female, 44.3% male, 0.1% unknown
- Race: 40.2% White, 48% African American, 5.2% Hispanic or Latino, 1.9% Asian, 4.7% Other
- Age: 30.1% were 18–50 years of age, 56.5% were 51–90, 13.4% were outside of those ranges

Invitations were sent to 1,000,740 diverse patients aged 18–90 to use a COVID-19 focused chatbot. The initial chatbot invitation was sent on March 25, 2020 and our analysis included patient interaction with the chatbot between March 25, 2020, and May 15, 2020. The invitations were sent to all patients that had activity (e.g. any kind of visit) with the healthcare system in the last year and had a documented mobile phone number or email address. Invitations were sent by text message if a mobile phone number was available. Email invitations were sent to patients that did not have a documented mobile phone number but had an email. Overall, 98.64% of the invited patients were sent text messages. The text message invite read, “{Name of healthcare provider organization} is providing a free COVID-19 symptom screener and education tool for our community. Get started. Reply STOP to opt out”. The email invite was the same without the “opt out” sentence. A reminder was sent to patients 24 h after the initial invite if there was no response or opt out in the case of the text message invites. The reminder text message read, “{Name of healthcare provider organization} can answer your COVID-19 questions and help you assess your risk. Get Started. Reply STOP to opt out”. The email reminder message was the same without the “opt out” sentence. The chatbot was available in English only and English was listed as the primary language in the EHR for 97.01% of invited patients.

The chatbot provided a COVID-19 symptom screener and learning module that included information on coronavirus, infection, testing, and recovery. Users could elect to not interact with the chatbot at all, interact with one of the modules, or both modules. We defined patient interaction as acceptance of the terms and conditions and use of some aspect of the chatbot, and module use as a patient initiating a chat within that module.

### Statistical analysis

For all patients invited to use the chatbot, their gender, race, and age were extracted from the EHR for subgroup analyses. Our analysis focused on the patient’s first use of the chatbot and did not include repeat use. Overall patient interaction rates, as a percentage, were compared using a two-sample *t*-test. Chi-Square tests for independence were used for sex, race, and age subpopulation statistical comparisons. Statistical significance was measured at the 0.05 significance level. Pairwise comparisons with Bonferroni corrections were used to compare the multiple levels of race. This study was determined to be exempt by the MedStar Health institutional review board and need for informed consent was waived. Data analysis was conducted using SAS software, Version 9.4 of the SAS© System.

### Reporting summary

Further information on research design is available in the Nature Research Reporting Summary linked to this article.

## Supplementary information

Reporting Summary

## Data Availability

The analyzed dataset is available from the corresponding author upon request.
